# Socio-Economic Status May Associate Different Risk(s) with Early Childhood Caries (ECC) That Can Cause the Development of Psychomotor Deficiency in Preschool Children Aged 3–6 Years Old: The Results of Preliminary Analysis from a Cohort Study

**DOI:** 10.3390/ijerph18179011

**Published:** 2021-08-26

**Authors:** Andy Yen-Tung Teng, Chen-Yi Liang, Yen Chun Grace Liu

**Affiliations:** 1 Center for Osteoimmunology and Biotechnology Research (COBR), School of Dentistry, College of Dental Medicine, Kaohsiung Medical University (KMU) & KMU-Hospital, Kaohsiung 80708, Taiwan; andytengyt@yahoo.com; 2 Laboratory of Molecular Microbial Immunity, Division of Periodontology, The Eastman Institute for Oral Health (EIOH), School of Medicine & Dentistry, University of Rochester, Rochester, NY 14620, USA; 3 Department of Childhood Education and Nursery, Chia Nan University of Pharmacy & Science, Tainan 71710, Taiwan; divide@so-net.net.tw; 4 Center for Osteoimmunology and Biotechnology Research, Department of Oral Hygiene, College of Dental Medicine, Kaohsiung Medical University, Kaohsiung 80708, Taiwan

**Keywords:** socio-economic status (SES), early childhood caries (ECC) and dmft, psychomotor, development and CCDI, preschool kindergartners, longitudinal cohort

## Abstract

Background: We have recently shown that there is a positive correlation between severe caries and developing psychomotor deficiency in preschool children. To fully re-assess such a relationship, we embarked on a 3-year longitudinal follow-up study of kindergarteners, where we aimed to: (i) confirm whether early childhood caries is causally related to the development of psychomotor deficiency as proposed, and (ii) address any significant role or contribution of socio-economic status associated with caries–psychomotor interactions in the preschooler family cohorts studied, over time. Methods: A longitudinal study was designed where the total sum of 159 kindergarteners aged 3–6 from the central and southern regions of Taiwan were randomly selected and recruited for clinical examination of caries, together with questionnaires for personal, demographic and dietary information, socio-economic status, and the children’s psychomotor development scales which were collected and analyzed over time. Student’s *t* test, chi-squared test, correlation coefficients, and multiple linear regression analysis with R^2^ determinants were employed to assess any attributable differences (of 0~1) between SES vs. psychomotor manifests and caries measured among all variables computed. Results: The results of our preliminary analyses show that: (i) there was likely a causal relationship between caries activities and aspects of general development scale via the Chinese Child Development Inventory over time (4.01 ± 3.47 vs. 5.88 ± 2.58, respectively) in the 3–6-year-old preschoolers, and (ii) there was significantly more attributable influence (via higher R-squared values) from SES and psychomotor manifests than that of caries and the Chinese Child Development Inventory counterparts, as detected over time. Conclusion: Collectively, the resulting analyses support our previous findings and confirm that there is likely a causal relationship between severe caries and psychomotor deficiency in growing preschoolers; the resulting analyses revealed that such causally related interactions may be attributably explainable by a content-reliant association via socio-economic status analyzed in the kindergartener family cohorts studied. Thus, the socio-economic status or its constituents/factors will have a much broader influence not only associated with developing early childhood caries (a biologic trait), but also for psychomotor deficiency (a social trait) in vulnerable children at risk.

## 1. Introduction

Early childhood caries (ECC) is the primary cause of toothache and tooth loss, which can be arrested early before becoming a chronic disease with irreversible consequences [1,2,3,4,5,6]. The sequelae may produce reduced occlusal forces on mastication, neurologic vs. brain stimulations, or impacts on physical metabolism and/or intellectual growth for developmental functions [5,6,7,8,9].

We have recently demonstrated that there is a positive correlation between severe ECC (dmft > 3~8) and psychomotor deficiency (i.e., expressive language and comprehension conceptual and general developmental scale, etc.) in regular/normal classes of kindergarteners (i.e., no developmentally delayed children involved) analyzed via a cross-sectional bi-township design [10,11,12]. Furthermore, other studies in the field have shown that the socio-economic status (SES) has long been considered one of the risk factors or even a predictor for ECC development and/or progress in children, including the levels of family/household incomes, parental education and occupation, access to medical/dental healthcare facilities, etc., all of which may be attributed to the equity issue of caries distribution in the population studied [13,14,15,16,17,18,19,20,21,22,23]. Likewise, SES may also be a domain for the oral health behavior and conceptual access to dental healthcare of the parents and/or caregivers in preschoolers [14,15,16,17]. However, additional risks suggested for ECC may include biofilm-associated microbial species, improper feeding, dietary or food practices, ethnicity/race, poverty-income classes, environmental/salivary vs. self-image attributes, and maternal psychosocial (i.e., sense of coherence) distress or parental stresses, etc. [15,17,18,19,20,21,24,25]. 

Further to studying the different attributes of associations with ECC on the preschoolers’ general health, we have been interested in assessing how ECC and related risks may be implicated in psychomotor deficiency, especially comprehension concepts, expressive language, or general developmental scales, etc., which remain unclear to date [10,11,12,24]. Herein, we have aimed to investigate whether ECC, via dmft measured, is causally associated with a child’s psychomotor deficiency or not (via CCDI: Chinese Child Development Inventory, modified by Hsu et al. [26]), with which a longitudinal cohort study was designed to analyze the 3–6-year-old kindergarteners randomly selected and recruited from central and south regions of Taiwan, as reported previously [10,11,12]. We also planned to address any significant role or contribution of SES to ECC and psychomotor manifests over time. As a result, our preliminary analyses showed that there was likely a causal relationship between ECC and the manifests of psychomotor deficiency (i.e., the general development scale of CCDI) in kindergarteners aged 3–6 years, and such interaction appeared much more attributably explained by the SES detected than by the caries measures (i.e., dmft) in those preschooler family cohorts analyzed. Therefore, modifying factors depicted into our present theme of hypothesis are discussed further.

## 2. Method and Materials 

The present study was designed to confirm whether ECC, via dmft, is causally related to a child’s psychomotor deficiency via CCDI scales, where a longitudinal cohort study was designed to analyze 3–6-year-old kindergarteners randomly selected and recruited from the central and south regions of Taiwan over time, as proposed [10,11,12]. In parallel, we analyzed any different role or contribution of SES to childhood caries (i.e., dmft) and their physical and psychomotor development in kindergartener family cohorts randomly selected and recruited as outlined above. Based on our recent reports [10,11,12], the protocols employed for study were herein directed via the same measures and criteria previously published, which are shown below regarding their details to achieving the objective as described above. 

### 2.1. Subject Selection

This project was first approved by the IRB and Ethics Committees of Kaohsiung Medical University, Kaohsiung, Taiwan, after which informed consent was obtained after the protocol had been thoroughly explained to the parents or legal guardians by the trained interviewers. For the project’s sample size estimate, we applied the published data using the “National Dental Survey of Children under Six of Taiwan” [27], where the sample size estimated is “334” (from south vs. central regions of Taiwan; each N = 170 × 2 = 340) to reach statistical significance, via the criteria selected as: α = 0.05, SD = 5.5, power = 0.9 and a 30% drop-out rate [12]. We recently reported the results of cross-sectional bi-township analyses from randomly selected cities of southern and central regions of the country (i.e., the southern, northern, eastern and central regions, as appropriate), and we adopted such random selection protocol in the same way, accordingly [10,11,12]. In addition, our past reports [10,11,12] and the present investigation looked to analyze preschoolers who all came from regular/normal classes of kindergarteners in their respective programs without any developmentally delayed or disabled children involved; thereby, the present guideline for enrolment of the 3–6-year-old children recruited for our study did not employ any specific inclusion vs. exclusion criteria listed in the original IRB protocols approved. 

To this end, we included the ≥3-year-old kindergartens, on a “first-come, first-served” basis for subjects’ recruitment and sampling (30% drop-out estimated), randomly selected from both south vs. central regions, which were known to exhibit different levels of dmft activities (~4.07 vs. ~6.88, respectively [10,11,12]), compared to that of our national average (~4.35; [27]). All preschool ≥3-year-old children consented and recruited went through the same clinical oral examinations and surveys, where their age, dmft scores, gender, BMI and food/diet intake, parental education vs. occupation, questionnaires of psychomotor developments (via CCDI) and behaviors were completed by the care-givers and/or teachers and then entered into the database for study. These approved procedures were performed at dual time points; firstly, when the children were ≥3 years old after their entry into the project (as the base-line), and later by or before the age of 5.5–6 years old (as the follow-up second time point), sequentially. For the present preliminary analysis, a total of 159 3–6-year-old preschool children (e.g., completion rate was 159/340 = 46.8%; 64 from the southern region vs. 95 from the central region) completed both the baseline and follow-up clinical exams and parallel surveys (e.g., follow-up period ranged 1.2~1.5 years from base-line; average participation rate 94.34%; central: 89.29% and south 100%).

### 2.2. Oral Examinations for the dmft Score

Clinical oral exams were performed based on the WHO guidelines (WHO 1997; oral health surveys: 4th Edition). Each participant received oral examinations from two calibrated dentists, whose resulting diagnostic reproducibility was estimated as κ = 0.80–0.84 [12], based on the same WHO guidelines. The oral examinations included the number of decayed, extracted, and filled primary teeth, where the sums of these measures were computed for the dmft scores, accordingly. 

### 2.3. Assessment of the Psychomotor Development and CCDI

The CCDI (Chinese Child Development Inventory), as modified by Hsu et al. [26] for post-adaptation from the Minnesota Child Development Inventory (MCDI, 1978; [26,28,29]), has frequently been used to assess children’s and infants’ physical and mental development [26,28,29]. The aspect scales of the CCDI included a total of 320 items on the list over seven developmental aspects in areas (i.e., gross motor, fine motor, expressive language, comprehension conceptual, situation comprehension, self-help, personal–social) and one overall summary scale (as the general development). Therefore, it is suitable for infants of 6 months up to 6-year-old children, including a questionnaire completed by the caregiver. In addition, the validity of the CCDI has been adequately appraised, where its correlation coefficients for all developmental areas analyzed were above 0.90 and its general development area was 0.837 [28,29]. Furthermore, the CCDI has also been shown to be useful for assessing children who may have development delays, with strong reliability (up to 0.94; [26,28]), whereas its reliability of all test aspects employed was estimated at 0.934, and the mean score of differences between the pre-test and post-test was estimated at 1.61%, ranging from 0.62% to3.75%; data not shown. Despite this, it has been estimated that the correlation of the CCDI with the Draw a Person (DAP) test is as good as 0.70 and that of the Chinese version of the Denver Developmental Screening Test is higher than 0.70 [26,28]. 

### 2.4. Acquisition of the SES Information and Data

With the approved IRB protocols, acquisition of personal and family information regarding children’s paternal vs. maternal education and occupation came from the surveys via questionnaires completed by the parents and/or legal guardian to document the subjects’ family status, which was standardized protocols of the Health Survey, Taiwan [12,30]. Meanwhile, the source of general information vs. statistics regarding the household/family incomes in the town, city, region and the national GDP level were derived from the database of the Ministry of Internal/General Affairs, and all of the healthcare-related information, including the frequency of accessing national health insurance programs for the medical, dental and drug/prescription services on the registrations and their usages for the applied statistics were from the official registry of the Ministry of Health and Welfares of Taiwan, as publicized and listed, respectively, at websites as follows: http://ebas1.ebas.gov.tw/pxweb/Dialog/statfile9.asp. [31] (accessed on 22 June 2021) and https://www.stat.gov.tw/ct.asp?xItem=968&ctNode=513 [32] (accessed on 22 June 2021). In addition, the parental educational and occupational classes employed for the present SES analyses were derived from the official records of the Ministry of Internal/General Affairs of Taiwan, where the published documents are cited in the references listed [33,34,35,36].

### 2.5. The Statistical Analyses

In the present study, all datasets were analyzed using the IBM computing software SPSS-Statistics (SPSS 22, IBM Corporate headquarter, released 2013; 1 New Orchard Rd., Armonk, NY 10504-1722, USA). The level of statistical significance was set at *p* < 0.05, where Student’s *t*-test was employed to compare variables, including caries (i.e., dmft) and CCDI measures among all subjects from the south vs. central regions. The chi-squared test was used to compare parental education vs. occupation (as part of SES analyses) among all subjects, and the correlations among the CCDI aspects (measured as the dependent variables), SES (i.e., parental education and occupation, the registered family/household incomes), averaged access to national health (medical and dental) care services vs. facilities, and caries (dmft; set as the control independent variables) were computationally analyzed. Thus, a multiple regression model was employed to analyze the relationship amongst all variables addressed in the study, with the R^2^, coefficient of determination (R squared [37]), the analysis correlations in a longitudinal cohort setting were employed to evaluate and model the interactive differences (0~1) between the key variables under investigation for attributable explanations, indicating the analytics applied to resolve how differences in one variable (i.e., SES) can be explained by a difference in a second or other variable(s) (i.e., dmft).

## 3. Results

A total of 159 kindergarteners recruited from central (*n* = 95) and southern (*n* = 64) Taiwan (out of the original 169) were enrolled in the present cohorts, having completed both clinical examinations and questionnaires/surveys, and were included in our preliminary analyses (i.e., 83 males: 52.2%; 76 females: 47.8%), and their dmft scores, SES and CCDI manifests are summarized in Table 1. Meanwhile, for SES, the parental education vs. occupation, documented family/household incomes, and the frequency of access to national healthcare (e.g., medical and dental) and facilities per person were also enlisted for the analyses. The results showed that the mean caries (dmft) score regarding ECC was 4.01 ± 3.47 and 5.88 ± 2.58 for the central vs. southern region, respectively, which was rather different from each other (*p* = 0.0004), consistent with the previous reports [10,11,12].

Furthermore, there was a significant difference observed between the CCDI manifests (i.e., situation comprehension and general developmental scale) and mean dmft scores between the central and southern regions (Table 1); a significantly detectable difference was found between the general development scale of CCDI aspects and the dmft scores (*p* = 0.0408) collectively measured over time from both regions in the present cohorts analyzed (see Table 1). Together, the above results detected from such follow-ups (e.g., between Central and South, and across the first and repeated second measures over time) suggest that ECC is likely a causal factor of developing CCDI deficiency over time based on the present analyses, during which there may have been physical changes in the children’s dentitions along with certain psychomotor deficits in progression as the function of time. Importantly, these new findings are consistent with our previous discovery using a cross-sectional bi-township design for study [12].

Furthermore, the resulting analyses showed that there were significant differences between certain CCDI manifests (i.e., gross motor (*p* = 0.0318) and personal-social (*p* = 0.0491)) and documented maternal education and occupation but not of the paternal kinds assessed. Upon step-wise comparisons, it was shown that there was a significant difference between CCDI aspects (i.e., situation comprehension (*p* = 0.0114) and general development scale (*p* = 0.0056); Table 2) and household income vs. the averaged frequency of access to national healthcare and facilities based on the documented data established (Table 2), suggesting that the SES may be significantly associated with developing certain manifests of CCDI deficiency in a timely fashion. Moreover, it is evidently clear that SES is likely to significantly associate different risks(s) with the development of psychomotor deficiency vs. caries activity over time in the present kindergarteners’ cohorts analyzed (see Table 1 and Table 2).

Intriguingly, when the regression analysis was applied via R^2^ modeling (R-squared) for the analytic correlations in a longitudinal setting to evaluate and assess any potential correlations between caries (via dmft) and SES measures (as the independent variables) towards the CCDI aspects, it was found that the levels of attributable difference (with higher R-squared values) were much more significant between SES and CCDI manifests (all aspects) than that between caries (dmft) and CCDI counterparts in the present cohorts analyzed (where *n* = 159; Table 3). Moreover, when such R-squared analysis was employed using the datasets of caries, SES measures, and CCDI aspects from our prior cross-sectional bi-township analyses (*n* = 353~433; [12]), a similarly higher level of significant difference was also detected between SES and CCDI manifests than that between SES and caries activities (see Table 3), indicating that the SES may likely play a significantly more attributable difference in mounting explainable associations with the risks of developing psychomotor deficiency in the growing preschool kindergarteners studied.

## 4. Discussion

Physical development in children involves the psyche-mental aspects during their growth, where good oral health is essential to achieving overall health and general well-being. ECC, if not treated properly, can progress into one of the most prevalent and costly diseases throughout the lifetime. Children experiencing ECC are likely to develop a greater probability of subsequent caries in both the primary and permanent dentitions, whose associated risks are complex and are also often compounded by additional biological vs. social variables (i.e., parental educations and SES, etc. [25]). Modern research on caries has indicated that the non-dental risks of ECC may include a low level of SES, other vulnerable sub-populations with disparities, and/or certain minority ethnical vs. racial groups, etc. [13,14,15,16,17,18,19,20,21,22,23,24,25]. Therefore, there is an urgent need to identify the critical risks of ECC and also target the high-risk sub-populations on prevention and effective protocols for subsequent proper treatments vs. managements. In this way, the overall financial and social costs can be further reduced for improvements. 

Our group recently reported that severe ECC (dmft > 3~8) is significantly associated with psychomotor deficiency (i.e., comprehension conceptual and expressive language, etc.) via cross-sectional bi-township analyses of randomly recruited preschoolers [10,11,12]. Notably, these preschoolers came from regular/normal classes of kindergartens without any developmentally delayed or disabled children involved, suggesting that when ECC, once started, progressively develops in normal growing children, it may trigger certain psychomotor deficiency in those vulnerable and at risk [12]. Thus, this new finding prompted us to launch a 3-year longitudinal study to reassess whether ECC is causally related to psychomotor deficiency, in the same randomly selected regional family cohorts of kindergarteners aged 3–6 years old, from which we addressed any additional contribution of SES regarding its associations with psychomotor manifests and caries activity measured over time (i.e., the social vs. biologic trail or indicator), via the multivariate analyses of the kindergarteners recruited in the present study. 

Interestingly, the results of present analyses confirm, for the first time, that there is indeed a causal relationship between ECC and psychomotor deficiency in growing children aged 3–6 years old, based on the recruited preschoolers that were measured twice and analyzed over time in the study. It is worth noting that maternal education and occupation, but not paternal metrics, were significantly associated with the gross motor and personal–social CCDI aspects (Table 2), a finding consistent with most of the reported results, emphasizing the critical maternal influence under the content-gradients of SES on ECC for oral health conditions evaluated [17,18,23,24]. 

**Figure 1:**
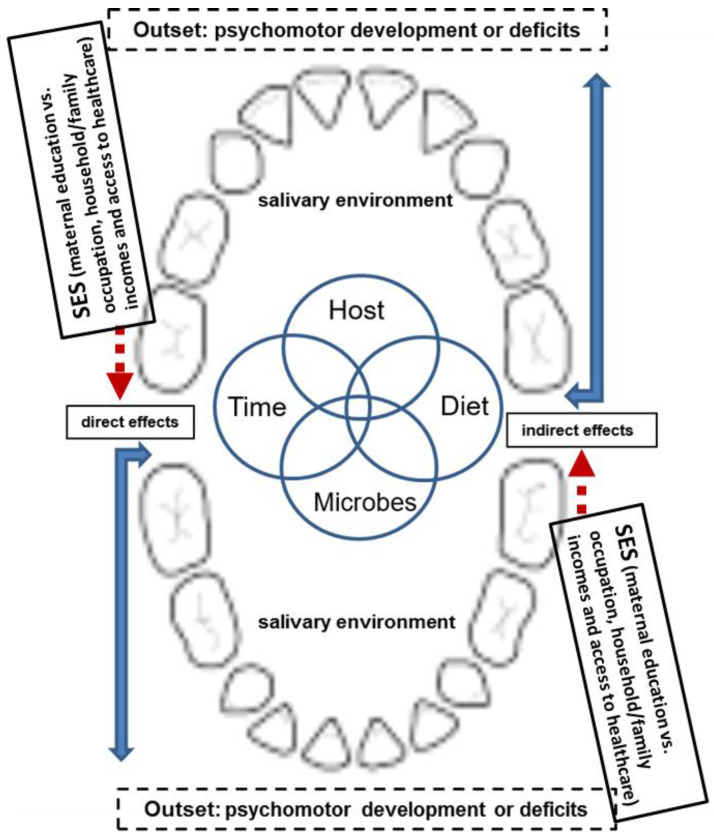
The SES is edited into the hypothetical framework of ECC-Psychomotor (development) axis proposed. Note: The SES is herein edited into the new hypothesis as we proposed recently (please refer to ref. [12]), where the content gradients of SES (i.e., the parental backgrounds, family incomes and access to healthcare facilities) are integrated during the initiation or progression of ECC caries (via dmft), in general.

Importantly, the collective results of multivariate and R-squared analyses (Table 2 and Table 3) indicate that the SES (i.e., maternal knowledge and attitudes, household incomes and utilization of healthcare services) not only significantly implicated the manifests of psychomotor deficiency, but also contributed more than the caries did in a timely manner. Moreover, this intriguing novel finding supports the caries–SES–psychomotor (development) axis that may exist to bridge the biologic vs. social association (or trait) on the risk(s) of ECC that can cause the development of psychomotor deficiency as a function of time [37,38,39,40]. Although there were comparable levels of SES measures in the present cohorts analyzed, psychomotor deficiency cannot be solely attributed by their social characteristics/trait in SES described (Table 1), consistent with our previous report [12]. Conceivably, such outcomes may likely arise through the critical stages of growth via personal language communications and psycho-social engagements as well [10], from which we have further implemented the different role or contribution(s) of SES onto the hypothesized theme proposed recently [11,12], consisting of a direct neurophysiologic path and/or an indirect one external to the neurologic circus (see Figure 1 and its legend).

The ECC–psychomotor axial interactions are complex, where the causal factors, risks, confounding variable(s), or interference may be intertwined [10,11,12,13,14,15,16,17]. Notably, there were three distinct characteristics in our present cohorts analyzed (Table 1): (i) there were insignificant healthcare inequity issues involved, due to the national healthcare insurance program available; (ii) the kindergartener family cohorts had annual income levels of the middle class, based on the SES gradients of the regions addressed; and (iii) the kindergarteners recruited and enrolled for the study all came from the regular/normal classes of preschoolers without any special or disabled children involved.

In our prior bi-township analyses (*n* = 353 & *n* = 433), it was found that there was no significantly mountable difference between the relevant independent variables (e.g., dmft vs. caries prevalence, age vs. gender (M/F), height vs. weight, BMI vs. distribution across all BMI categories, and intakes vs. frequencies of food categories; [10,12] ); as inversely opposed to the developmental quotients (DQ) detected for the resultant significant associations with CCDI scales (as the dependent variables), suggesting the changes in a child’s sequential development and/or deficiency, as a function of time (i.e., age [12]). Furthermore, concerns about the confounding variables or interference with dmft measured during psychomotor development have been assessed and statistically rectified via the Pearson and partial Pearson correlations and multiple regression analyses, yielding insignificantly much lower associations with such influence from confounding variables [10,12]. 

Conceivably, employing the kindergarteners randomly recruited from the same central and southern regions (*n* = 159), parallel regression analyses of relevant independent variables vs. dependent measures in our present cohort study have yielded minimally mounted levels of interference from confounding variables (i.e., gender, age, height and weight, and BMI, etc.), significantly less than those of SES and caries did to the CCDI manifests overall (data not shown here). Moreover, it remains to be seen further whether such analytic outcomes and interpretations will be in concordance with the above prior analyses on the confounders’ issue, which is a key limitation of the present analyses from our cohort study. Additional limitations of the present study may also include: the parent–child relationship and parenting qualities that may interfere the oral health and psychomotor determinants with the development of verbal vs. language skills; additionally, any unfeasible or uncontrollable factors that may complicate the conditions of SES indirectly, such as environmental stress or personal psychosocial coherence [20,21,23,24,25].

Upon further investigations through our analysis correlations in the longitudinal cohort setting, it is hoped that the ECC–SES–psychomotor axis revealed will aid to testify the hypothesis proposed (Figure 1, and [36,37,38]). With regard to how ECC may trigger sound distortion, misarticulation, or poorer oral functions, such as physical malocclusion [2,3,4,10], and deficient language development for maturation [12], these must be further investigated before any definitive conclusion is drawn. In addition, our proposed hypothesis-driven theme may also be applied to evaluate kindergarteners with special needs, disabilities or systemic illnesses to counterbalance the CCDI features for furthering the outcome analyses; despite that there is no such special cohort of children involved in the present study, as noted above. Finally, once fully revealed, the confirmed ECC–psychomotor causal interactions [12] and the suggested ECC–SES–psychomotor axis revealed in the present study may become a well-perceived mechanistic framework beyond what has not been anticipated or realized in the past. By then, any critically rectified means of strengthening the maternal influence and deploying financial/fiscal implementations through the content-gradients of SES determinants will enable us to achieve a significant improvement of ECC for better oral health and related sequelae for our preschool children.

## 5. Conclusions

Based on our recent reports [10,11,12] and the present findings, ample evidence is presented to confirm that, firstly, ECC is one causal factor for developing psychomotor deficiency in growing preschoolers over time most likely, with which, secondly, it is unforeseeably shown that such causally related interactions be significantly and attributably explained by different risk(s) via the SES–psychomotor association in the preschooler family cohorts studied. Importantly, the biological influence of SES determinant(s), when fully substantiated upon completion of the present analysis correlations in a longitudinal cohort setting, with the ECC–psychomotor axis will be much deeper than anticipated to date, needless to mention the accompanying wider social and social–economic influence(s). Further understanding of the underlying intricate interactions among the critical issues (i.e., the ECC–SES–psychomotor axis), during the child’s development will likely facilitate us in establishing better oral healthcare programs and prevention strategies to aid the wellbeing and overall fine development for our young children in the long run.

## Figures and Tables

**Table 1:** The significant association between caries (dmft) and certain CCDI manifests vs. the SES categories for the present cohorts studied.

	South (*n* = 64)		Central (*n* = 95)				
**Gender (M/F Number)**	39/25		44/51				
**Caries (dmft: Mean ± SD)**	5.88 ± 2.58		4.01 ± 3.47		South + Central Regions		
					Caries vs. CCDI (Correlation Coefficient)		
**CCDI Aspects**					Related Coefficient	*p*-Value	*n*
Gross motor	88.22 ± 26.44		92.16 ± 18.08		−0.0035	0.9670	141
Fine motor	100.11 ± 15.01		100.83 ± 5.84		−0.1246	0.1453	138
Expressive language	98.46 ± 14.86		98.30 ± 3.13		−0.0059	0.9449	138
Comprehension conceptual	113.43 ± 19.02		112.09 ± 7.76		−0.0201	0.8153	138
**Situation comprehension**	95.80 ± 16.06		100.68 ± 5.84 *		−0.0458	0.5936	138
Self-help	96.02 ± 16.33		97.53 ± 7.08		−0.0991	0.2477	138
Personal–social	93.81 ± 15.71		97.40 ± 7.19		−0.0763	0.3739	138
**General development scale**	103.71 ± 15.66		108.76 ± 4.25 *		−0.1744 *	0.0408 *	138
**Paternal educations**	number	%	number	**%**			
Primary/elementary school or below	1	1.56	1	1.05			
Junior high school graduate or below	2	3.13	2	2.11			
High school or vocational graduate/equivalent	26	40.63	25	26.32			
Associate degree/college or university graduate	32	50.00	49	51.58			
Graduate school/institute and/or above (Master or Doctoral)	3	4.69	15	15.79			
**Paternal occupations**							
Unemployed and non/semi-skilled workers	20	31.25	27	28.42			
Skilled professional workers	21	32.81	25	26.32			
Semi/full professionals, public servants, heads of small business	19	29.69	26	27.37			
Professionals, mid-level executives and above, corporate leaders	4	6.25	16	16.84			
**Maternal educations**							
Primary/elementary school or below	0	0.00	0	0.00			
Junior high school graduate or below	3	4.69	2	2.11			
High school or vocational graduate/equivalent	19	29.69	28	29.47			
Associate degree/college or university graduate	41	64.06	55	57.89			
Graduate school/institute and/or above (Master or Doctoral)	0	0.00	6	6.32			
**Maternal occupations**							
Un-employed and non/semi-skilled workers	29	45.31	37	38.95			
Skilled professional workers	16	25.00	19	20.00			
Semi/full professionals, public servants, heads of small business	15	23.44	23	24.21			
Professionals, mid-level executives and above, corporate leaders	3	4.69	13	13.68			
**Family/household incomes/year (USD)**	**43,770**		**33,628**				
**Frequency of accessing the health-care and facilities/person**	**1.61**		**1.30**				

* *p* < 0.05. Note: The source information about the SES was derived from the records of the Ministry of Internal/General Affairs and the Registry of Health and Welfares of Taiwan, as cited in refs: [31,32] shown in references below.

**Table 2:** The statistical analyses for the significance between the SES measures and certain CCDI manifests.

CCDI Aspects	Paternal Education			Maternal Education			Paternal Occupation			Maternal Occupation			Family/Household Income			Frequency of Accessing Healthcare and Facilities/Person		
	Related Coefficient	*p*-Value	N	Related Coefficient	*p*-Value	N	Related Coefficient	*p*-Value	N	Related Coefficient	*p*-Value	N	Related Coefficient	*p*-Value	N	Related Coefficient	*p*-Value	N
Gross motor	0.1055	0.2004	149	0.1766 *	0.0318	148	0.1519	0.0635	150	0.1129	0.1717	148	−0.0886	0.2792	151	−0.0886	0.2792	151
Fine motor	0.0468	0.5804	142	0.1063	0.2097	141	0.1478	0.0782	143	0.1204	0.1550	141	−0.0332	0.6928	144	−0.0332	0.6928	144
Expressive language	−0.0695	0.4112	142	0.0095	0.9113	141	0.1132	0.1784	143	0.0650	0.4437	141	0.0077	0.9274	144	0.0077	0.9274	144
Comprehension conceptual	−0.0875	0.3002	142	0.0485	0.5682	141	0.1105	0.1889	143	0.1342	0.1126	141	0.0488	0.5615	144	0.0488	0.5615	144
Situation comprehension	0.0574	0.4976	142	0.0806	0.3421	141	0.1542	0.0660	143	0.1197	0.1574	141	−0.2103 *	0.0114	144	−0.2103 *	0.0114	144
Self-help	−0.0362	0.6692	142	0.0488	0.5659	141	0.0871	0.3011	143	0.0915	0.2808	141	−0.0635	0.4493	144	−0.0635	0.4493	144
Personal–social	−0.0363	0.6679	142	0.0186	0.8265	141	0.1470	0.0797	143	0.1660 *	0.0491	141	−0.1530	0.0672	144	−0.1530	0.0672	144
General development scale	−0.0023	0.9783	142	0.0582	0.4931	141	0.1296	0.1228	143	0.1409	0.0956	141	−0.2299 **	0.0056	144	−0.2299 **	0.0056	144

**, *p*< 0.01. *, *p*< 0.05.

**Table 3:** The R-squared analysis for any different attribute(s) on the CCDI manifest.

CCDI Manifest	South + Central Regions (*n* = 159; from the Preliminary Analysis of the Present Cohorts Being Studied)			South + Central Regions (*n* = 353~433; from a Cross-Sectional Bi-Township Analysis; See Ref. [12])	
	R-Squared			R-Squared	
	SES	Caries	SES + Caries	SES	Caries
Gross motor	0.0498	0.0000	0.0502	0.0098	0.0013
Fine motor	0.0346	0.0155	0.0952	0.0797	0.0309
Expressive language	0.0290	0.0000	0.0649	0.0786	0.0327
Comprehension conceptual	0.0550	0.0004	0.0941	0.1253	00.0498
Situation comprehension	0.0741	0.0021	0.0788	0.0815	0.0234
Self-help	0.0309	0.0098	0.0419	0.0993	0.0255
Personal–social	0.0764	0.0058	0.0454	0.0211	0.0042
General development scale	0.0841	0.0304	0.1104	0.0992	0.0366

## Data Availability

The data employed in the present report is available upon the email request to the correspondence and 1st authors, as listed (graceliuyc@gmail.com & andytengyt@yahoo.com).
